# Role of collagen and immunostaining for TGF-β in the clinical and microscopic findings of pyogenic granuloma and peripheral ossifying fibroma

**DOI:** 10.4317/medoral.26268

**Published:** 2024-01-30

**Authors:** Paulo Goberlânio de Barros Silva, Dayrine Silveira de Paula, Guilherme Costa Soares, Lirya Nágyla de Souza Cavalcante, Isabelly Vidal do Nascimento, Fabrício Bitu Sousa, Mário Rogério Lima Mota, Ana Paula Negreiros Nunes Alves

**Affiliations:** 1 Department of Dental Clinic, Division of Oral Pathology, Faculty of Pharmacy, Dentistry and Nursing, Federal University of Ceará, Fortaleza, Ceará, Brazil; 2 Department of Dental Clinic, Unichristus, Fortaleza, Ceará, Brazil

## Abstract

**Background:**

Collagen is a component of Pyogenic Granuloma (PG) and Peripheral Ossifying Fibroma (POF) and performs different functions in these lesions. The objective of this study is to evaluate the role of collagen and immunostaining for Transforming Growth Factor beta (TGF-β) in the clinical and microscopic findings of PG and POF.

**Material and Methods:**

PG (*n*=20) and POF (*n*=20) were selected for clinical evaluation (sex, age, localization, size and evolution time) and microscopic analysis (picrosirius red staining for collagen analysis and immunohistochemistry for TGF-β) performed in the superficial and deep areas of the two lesions. ANOVA/Bonferroni and t-test, Pearson correlation and χ2 were used to compare the sites and parameters analyzed (*p*<0.05, GraphPad Prism 5.0).

**Results:**

The depth of PG presented the highest amount of collagen (*p*<0.001), and its surface showed the lowest amount of type 1 collagen (yellow-red strong birefringence). Type 1 collagen gradually increased in depth of PG, surface and depth of POF (*p*<0.001). The number of TGF-β+ cells was lower on the surface of PG compared with the depth of PG and the two areas of POF (*p*<0.001). Sex and localization did not affect these parameters, but the profile of collagen and immunostaining for TGF-β suffered from modifications by the time of evolution and the size of the lesion.

**Conclusions:**

Although PG and POF are reactive gingival lesions, the expression of TGF-β and its role in collagen showed different biological behaviors in these lesions, suggesting different biological origins for its components.

** Key words:**Pyogenic granuloma, peripheral ossifying fibroma, collagen, transforming growth factor beta.

## Introduction

The proximity of gingiva with the alveolar mucosa, periodontal ligament, teeth and alveolar bone places this anatomical site at a higher risk for the development of specific and non-specific lesions. This attribute confers difficulty in understanding lesions of the gingiva (1). Epidemiological surveys of gingival lesions are frequently performed (2-4), but the methodologies are focused on documents and medical records surveys, making it difficult to create hypotheses of mechanisms of pathogenesis of different lesions.

The most frequent lesions in gingiva are reactive, and despite slight variations, the Pyogenic Granuloma (PG) and Peripheral Ossifying Fibroma (POF) are the most common (1,4-6). These two lesions are non-neoplastic, have nodular growth, can reach large sizes and are caused by chronic low-intensity trauma, periodontal disease and dental biofilm (1,7-9).

However, while the PG exhibits frequent bleeding (10), the POF is deeply hard and crackling (11). The PG exhibits intense vascular proliferation and chronic inflammation (4), and the POF has fibroblast proliferation associated with a mineralized material (bone, cementoid or dystrophic calcification) (12,13). In addition, the PG can be found in several oral sites (14), but the POF is exclusively found in gingiva (11).

It has been proposed that the POF and PG have different cellular origins (11), and the role of collagens and cytokines involved in development and maturation can help to explain this theory. TGF-β is a cytokine that is strongly associated with the production and maturation of collagen (15), which is an important component in these two lesions. Overproduction of TGF-β can lead to tissue growth (16), vascular proliferation (17), bone formation (18), and collagenesis, which has been demonstrated in POF and PG (13).

TGF-β is an important biostimulator of collagen synthesis, but its activity is modified in the presence of inflammation (present in PG) (19). However, the maturation of PG occurs in parallel with a decrease in inflammation (1), which is necessary for bone maturation (18) and begs the question of why PG does not evolve to POF.

Understanding the role of TGF-β in the collagen profile of these two lesions may help to understand their etiopathogenesis and evaluation. Thus, the objective of this study was to evaluate the role of collagen and immunostaining for TGF-β in the clinical and microscopic findings of PG and POF.

## Material and Methods

- Sample size calculation

Recently, Zhang (7) comparing clinical characteristics of PG and POF showed that prevalence of bleeding is higher in PG (92.74%) than POF (39.91%). Such blending is directly associated with ulceration on PG reinforcing MMPs and collagen degradation (7), we used this data for sample size calculation. So, a sample of 20 cases per group (α = 0.05; β = 0.10) was calculated using Fleiss method with continuity correction.

- Type of study and sample characteristics analyzed

This is a cross-sectional, quantitative and retrospective study in which 20 PG and 20 POF were selected from Oral Pathology Laboratory of Federal University of Ceará and all patients are diagnosed and treated in the same medical center. The histopathological reports contained clinical information of sex, age, localization, size and evolution time. The histological slides were descriptively analyzed, and paraffin blocks were of adequate quality and quantity of material for histological analysis. Patients with PG extra gingival, patients with more than one PG or POF lesion, specimens from pregnant women, and patients diagnosed with other lesions or systemic conditions were excluded. Ulcerated POF were also excluded.

- Histochemical and immunohistochemical reactions

Samples of tissues were cut into 3-µm-thick sections, which were then placed on slides and processed. The samples were deparaffinized, rehydrated and subjected to coloration with Picrosirius Red (Scytech®) (30 min). Briefly, the samples were washed in 5% HCl and counterstained with Harris Hematoxylin for 45 s followed by dehydration, diaphanization and mounting with Enthellam®.

Tissue samples were cut into 3-µm-thick sections, which were then placed on silanized slides and processed. Brieﬂy, the samples were deparafﬁnized, rehydrated and subjected to antigen-recovery using citrate buffer (pH 6.0) in heat (95 ºC) for 30 minutes. To inactivate the endogenous peroxidase, the specimens were incubated for 30 min with 3% H2O2 in PBS (Phosphate Buffer Solution) and incubated with primary antibody Anti-TGF-β (1:400, Abcam®) overnight. After washing in PBS, the samples were incubated in secondary antibody Histofine (Nicherei®) for 60 min and diaminobenzidine chromogen (Abcam®) was applied to the specimens for 5 min. Harris hematoxylin (10 s) was used as a counterstain. Thus, the specimens were dehydrated, diaphanized and cover-slipped using Enthellam®. A human lymph node was used as positive control of the reaction and negative control of the reaction was performed by omitting the primary antibody.

Six micro-fields of the surface and six depth micro-fields of depth of PG and POF were imaged using a microscope (DM2000, Leica®) equipped with a camera (DFC295, Leica®) and software Leica Application Suite (LAS, Leica®) in 400× magnification (Area = 1.56 mm²) for collagen analysis and quantification of TGF-β positive cells. The photomicrographs for collagen analysis were performed in common and polarized light.

- Microscopic analysis: optical density analysis of collagen fibers

The picrosirius images obtained under conventional light microscopy were analyzed using ImageJ® (NIH Image) software by the Color Threshold (Image > Adjust > Color Threshold) command in RGB function. We adjusted the color scales from red (minimum: 71, maximum: 255), green (minimum: 0, maximum: 69) and blue (minimum: 0, maximum: 92) from select red staining (collagens fibers). After calibration, the images were converted to 8-bit scale (Image > Type > 8-bit), binarized (Process > Binary > Make Binary), and the total area of collagen was measured (Analyze > Analyze Particles). The mean of three micro-fields of the superficial area and depth area was used as a sample unit (20).

- Microscopic analysis: Optical density analysis of collagen fibers in polarized light

The picrosirius images obtained under polarized light microscopy were analyzed in ImageJ® (NIH Image) software using the same command. The colors were adjusted by the Color Threshold (Image > Adjust > Color Threshold) command in RGB function to red (minimum: 0, maximum: 255), green (minimum: 0, maximum: 255) and blue (minimum: 0, maximum: 32) from select yellow staining (type I collagens fibers). After calibration, the images were converted to 8-bit scale (Image > Type > 8-bit), binarized (Process > Binary > Make Binary), and the total area of collagen was measured (Analyze > Analyze Particles). The mean of three micro-fields of the superficial area and depth area was used as the sample unit.

The yellow-red birefringence area (type I collagens fibers) was subtracted from the red area (total collagens fibers) to obtain the greenish birefringence area (type III collagens fibers). The mean of three micro-fields of the superficial area and depth area was used as the sample unit.

- Microscopic analysis: Immunostaining from TGF-β

Immunohistochemical images from TGF-β antibody were analyzed using ImageJ® (NIH Image) software by the Cell Counter command. A trained and calibrated operator quantified the TGF-β-positive and -negative cells to calculate the percentage of positive cells. The mean of three micro-fields of superficial area and depth area was used as the sample unit.

- Statistical analyses

Quantitative data (mean ± SE) were analyzed using the Kolmogorov-Smirnov test and compared using ANOVA/Bonferroni, t-test and Pearson correlation (parametric data). The clinical categorical data (n, %) was compared using χ2 or Fisher’s Exact test. We used the Graph Pad Prism 5.0 (GraphPad Software, Inc., California, USA) with a 95% confidence level.

## Results

- Characterization of the sample

Of the total of 20 cases of PG, thirteen were female (65%) and seven were male (35%). Of the total of 20 cases of POF, twelve (60%) were female and eight (40%) were male. There was no difference between sex distribution (*p*=0.744) (Table 1).

The maxilla had the highest prevalence of PG (*n*=14, 70%) and was statistically different for POF, which had the highest prevalence in the mandible (*n*=13, 65%) (*p*=0.027). The anterior zone had the highest prevalence of PG (*n*=14, 70%), which was statistically different for POF, which had the highest prevalence in the posterior zone (*n*=15, 75%) (*p*=0.004) (Table 1).

The mean age of PG patients was 35.4±3.8, which was statistically similar to POF (29.9±3.4) (*p*=0.325), and the mean of evolution time was similar in the two lesions (7.1±2.9 months and 8.7±3.0 months, respectively) (*p*=0.705). However, the mean size of PG (1.5±0.2 cm) was significantly lower than POF (2.4±0.3 cm) (*p*=0.016) (Table 1).

**Table 1:**
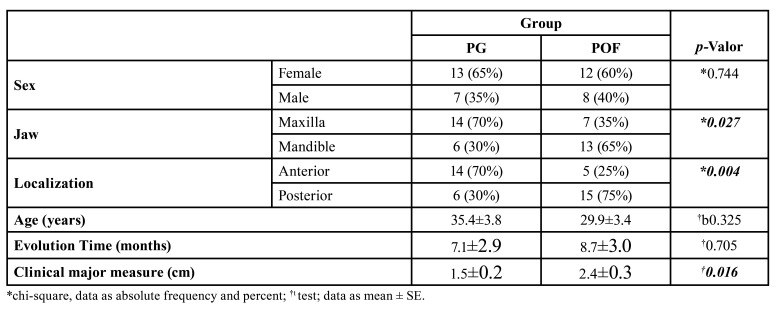
Clinical characteristics of PG and POF cases of Oral Pathology Laboratory of Federal University of Ceará.

- Microscopic Characterization of PG and POF

The PG showed an ulceration in the superficial zone with intense mixed inflammatory infiltrate consisting of polymorphonuclear neutrophils cells and mononuclear cells. A large amount of vascular proliferation in the middle of fibrous connective stroma consisting of thick collagen fibers was shown in the depth of PG.

The POF was completely covered by parakeratinized epithelium, under which there was dense connective tissue with mature blood vessels and rare inflammatory cells. In the depth of POF, a large number of ovoid cells were associated with mineralized material consisting of bone, cementum-like material or dystrophic calcification.

- Collagen profile of PG and POF

**Figure 1:**
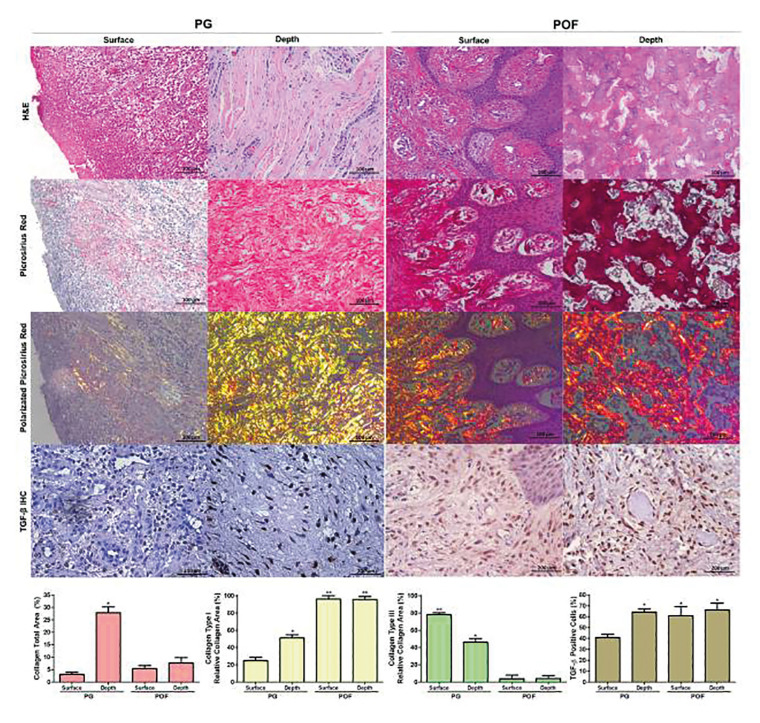
Histological, histochemical and immunohistochemical profiles of the superficial and depth areas of the PG and POF cases of Oral Pathology Laboratory od Federal University of Ceará (Magnification: 200; H&E: Hematoxylin-eosin; IHC: Immunohistochemistry). The microscopic analysis showed an intense inflammatory infiltrate, less collagen and immunostaining for TGF-β in surface of PG and less inflammation, high collagen deposition (type I) and immunostaining for TGF-β in depth of PG. In surface and depth of POF lower inflammation, high levels of type I collagen and immunostaining for TGF-β are showed. *p<0.05 versus surface of PG; **p<0.05 versus depth of PG (mean ± SE, ANOVA/Bonferroni).

The total collagen of the superficial area of PG (3.1±0.7%) was significantly lower than the depth area of the same lesion (27.8±2.4%). The depth of PG showed more collagen than the surface (5.4±1.2%) and depth of POF (7.6±2.6%) (Fig. 1).

Of the total area of collagen, the superficial area of PG (24.6±4.1%) showed the smallest area of yellow-red birefringence (collagen type I) compared to the depth of PG (51.1±3.8%). These values were significantly lower than the surface (96.0±4.0%) and depth (95.7±3.3%) of POF (*p*<0.001). In contrast with the total area of collagen, the superficial area of PG (77.9±2.3%) showed the highest greenish birefringence area (collagen type III) compared to the depth of PG (46.4±4.1%) and surface (4.0±3.6%) and depth (4.3±3.3%) of POF (*p*<0.001). (Fig. 1).

- Immunostaining of TGF-β of PG and POF

The PG showed the smallest number of TGF-β-positive cells in its surface (40.8±3.1%), which was significantly lower than the depth of the same lesion (64.2±3.1%) and surface (60.9±8.3%) and depth (66.2±6.4%) of POF (*p*=0.001) (Fig. 1).

- Clinical-pathological and microscopic correlation between the collagen profile and TGF-β immunostaining in different PG and POF zones

Sex and localization did not significantly influence the collagen profile and immunostaining of TGF-β in PG and POF (Table 2). However, in PG, the time of evolution was directly correlated with the type I collagen (yellow-red birefringence) in depth (*p*=0.042, r=0.422) and inversely correlated with type III collagen (greenish birefringence) in the same region (*p*=0.018, r=-0.583). In POF, the number of TGF-β-positive cells on the surface was directly correlated with the size of the lesion (*p*=0.028, r=0.972), and the number of TGF-β-positive cells in depth was inversely correlated with evolution time (*p*=0.019, r=-0.887) (Table 2).

In PG, the total collagen on the surface was directly correlated with type III collagen (greenish birefringence) on the surface (*p*=0.001, r=0.598) and in depth (*p*<0.001, r=0.682) and inversely correlated with type I collagen (yellow-red birefringence) on the surface (*p*=0.001, r=-0.598) and in depth (*p*<0.001, r=-0.682). Type I and III collagen were inversely correlated with both regions (*p*<0.001, r=1.000). Immunostaining for TGF-β did not show any correlations with the collagen profile in PG (Table 2, Table 3).

**Table 2:**
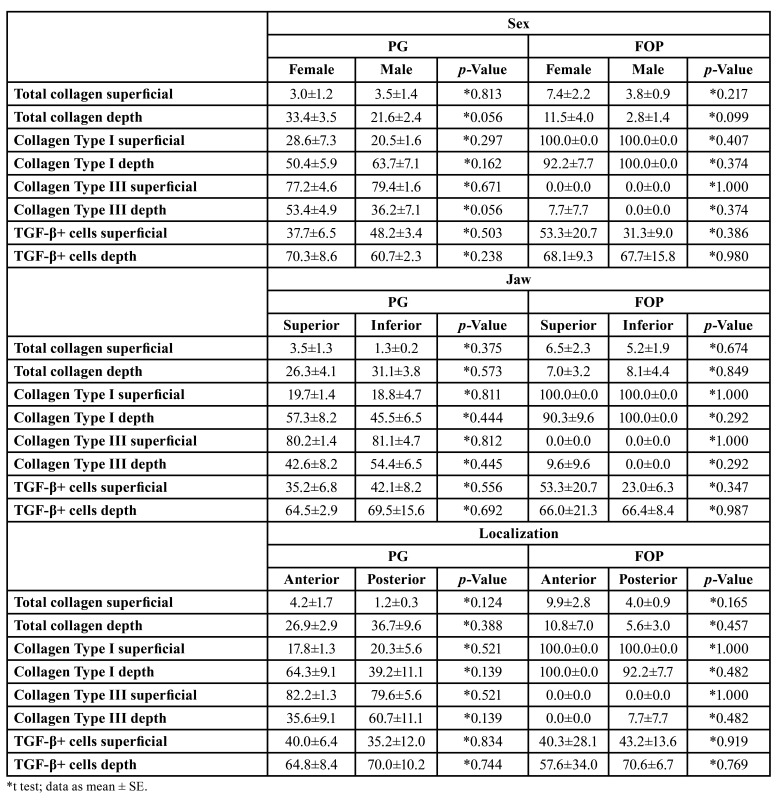
Effect of Sex, Jaw and Localization in the Histological Findings of PG and POF cases of Oral Pathology Laboratory of Federal University of Ceará.

**Table 3:**
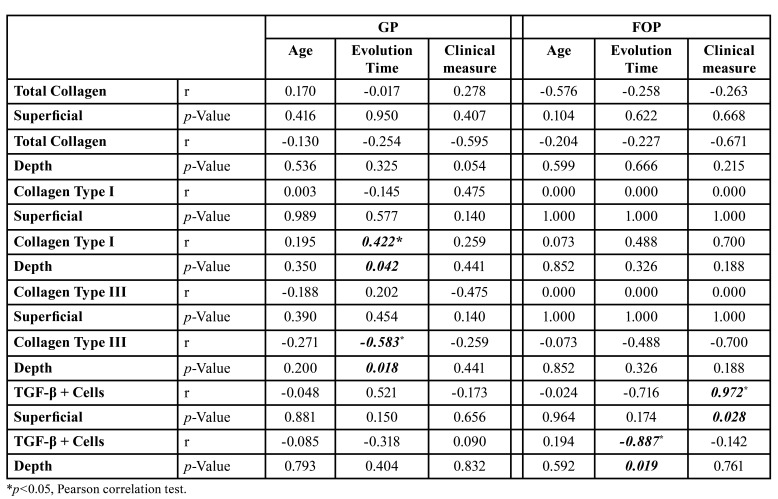
Effect of Age, Time Evolution and Lesion Measures in the Histological Findings of PG and POF cases of Oral Pathology Laboratory of Federal University of Ceará.

In POF, the total collagen on the surface showed a direct correlation with the total collagen in depth (*p*=0.027, r=0.633), and type I (yellow-red birefringence) and III (greenish birefringence) were directly correlated both surface and depth (*p*<0.001, r=1.000). TGF-β immunostaining on the surface of POF was directly correlated with the total collagen in depth (*p*=0.027, r=0.811), and TGF-β immunostaining in the depth of POF was directly correlated with type I collagen on the surface (*p*<0.001, r=1.000) and inversely correlated with type III collagen in depth (*p*<0.001, r=-1.000) (Table 2, Table 4).

**Table 4:**
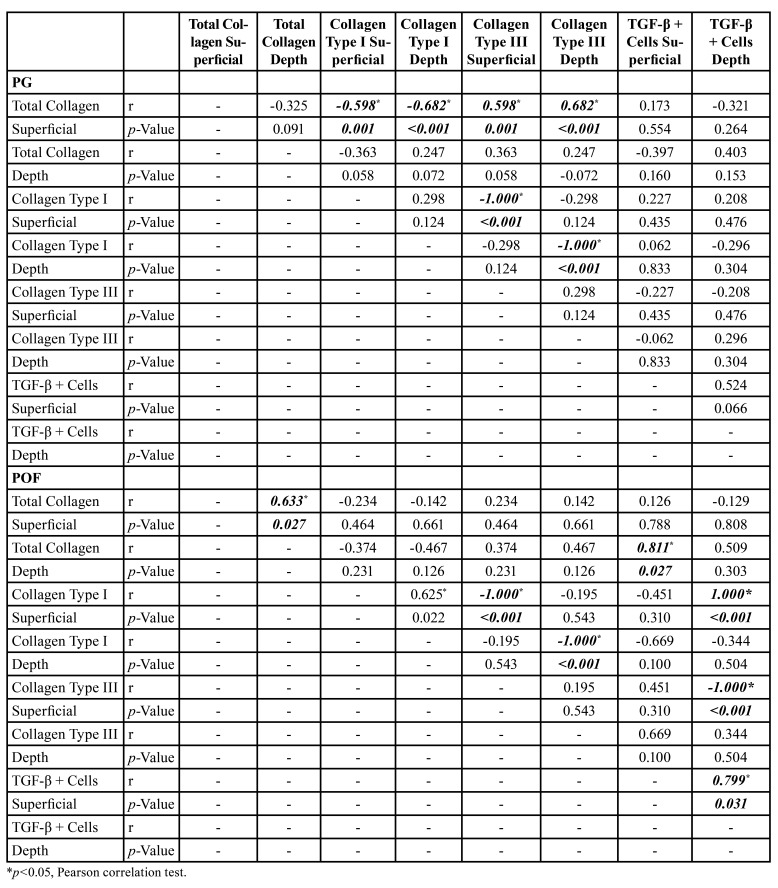
Correlation between TGF-β immunostaining and profile of collagen in the PG and POF cases of Oral Pathology Laboratory of Federal University of Ceará.

## Discussion

The PG and POF are two of the most prevalent lesions in gingival tissue, and they can be reclassified according to their origin. It has been proposed that PG is caused by pyogenic bacteria, and POF has a neoplastic origin (1). Currently, it is known that PG and POF are reactive lesions that are strongly associated with dental biofilm (3-4).

In the present study, epidemiological surveys did not show any significant differences between PG and POF distribution by age and predilection by women (4-6). The topographic distribution varies depending on the study, demonstrating that there are more cases for PG in the maxilla and anterior zone (7) and more cases for POF in the mandible and posterior zone (21), as shown in our study, although the opposite has also been described (6). It is worth highlighting the difficulty in performing location matching, considering that we work with a biobank with available samples. For this reason, as it was not possible to pair, location was compared as a confounding factor.

The size and evolution time are features that have been less well studied. While Zhang and colleagues (4) showed a longer evolution time of PG compared to the POF, our study showed no significant difference. In addition, although Effiom and colleagues (5) did not show any differences in the size of these two lesions, Kfir and colleagues (6) demonstrated that POF was significantly larger than PG (similar to our study).

Regarding the clinical and microscopic characteristics, the PG exhibits bleeding and is firm, nodular, red, and thickly inflamed and ulcerated (7) and the POF is firm and nodular, with specific ovoid cells, high cellularity, moderate vascularity and without inflammation (22). Despite these differences, PG and POF have rich stroma in connective tissue; however, the POF, but not the PG commonly, demonstrates mineralization (22-23).

The total collagen and type I collagen (matured form of collagen) gradually increased from the surface to the depth of the PG. The surface of the PG is intensely inflamed, and intense inflammation enhances the expression of matrix metalloproteinases (MMP). MMPs degrade collagen, reducing its quantity and thinning the fibers (24-25). Furthermore, the depth of PG exhibits high immunostaining for TGF-β, a cytokine that is strongly associated with the production and maturation of collagen (15). However, the depth of PG does not show high levels of inflammation. Thus, this may explain the overproduction and maturation of collagen in the depth of PG, which is common in old PG with mild inflammation (1).

In POF, there are low levels of total collagen, but the proportion of collagen type I is the highest, as well as the expression for TGF-β. TGF-β stimulates not only the production of collagen but also the maturation of type III collagen in type I collagen (26), and its ability has been demonstrated to modulate the production of bone matrix, particularly in the absence of inflammation (27). Previous studies have suggested that in some cellular types, the role of TGF-β is more strongly associated with collagen bone maturation than collagen overproduction (15,18,22). If this is true, then would PG cells be different from POF cells?.

Fibroblasts, cells present in the PG and POF, produce TGF-β; (15) however, the origin of these cells appears to be different in these lesions. Fibroblasts of periodontal ligament produce higher levels of osteoprotegerin (OPG) than gingival fibroblasts (28). OPG is a cytokine that leads to bone maturation, and thus, the origin of theses fibroblasts can be different. Kumar and colleagues (11) suggested that the fibroblasts of POF originated from gingival ligament, and thus, the PG fibroblasts may have originated from gingiva or connective tissue, justifying extra gingival PG (1).

Oral topography is not a factor related to collagen production. The increase in collagen production depends on the presence or absence of the traumatic factor, for this reason, Pyogenic granulomas that are related to the traumatic factor have less collagen (29,30).

Ulcerated lesions tend to express more metoloproteinases, due to the inflammatory process and because of this, pyogenic granulomas tend to have less collagen, consequently being less fibrous. On the other hand, Peripheral Ossifying Fibroma does not present an ulcer, consequently providing a more favorable environment for collagen production, which is why these lesions are more resistant and firmer to palpation (30). These characteristics help in the clinical diagnosis, as the granuloma is generally more flaccid and bleeding, causing loss of the epithelium. In contrast, Peripheral Ossifying Fibroma is firmer, as it produces more collagen (29-31).

Elanagei and colleagues (32) showed that, in contrast to PG, the cells in the depth of POF produces osteopontin (OPN), a protein responsible for the deposition of mineral matrix in the organic matrix (9) in the presence of TGF-β (33). Thus, these findings support our hypothesis that POF cells are of different origin from PG.

In addition, our findings suggest that POF growth and its collagen maturation are dependent upon TGF-β produced on the surface, but over long periods of time, there is a reduction in the depth of TGF-β production. A previous study (15) showed that the production of TGF-β is increased in the presence of LPS, and with enhanced POF size and the distance of center of lesion, the causal factor (biofilm) of TGF-β tends to reduce, self-limiting the size of the POF. Overall, we showed that in POF, the production of collagen is dependent on TGF-β, and the maturation of collagen is directly associated with the quantity of collagen. In PG, our data suggest that collagen maturation is directly associated with evolution time, supporting the hypothesis of fibrous maturation of PG, as suggested by STablein and Silverglade (1).

Thus, due to different compartments of collagenesis, we suggest that POF and PG are of distinct fibroblast cell origins. Due to the absence of inflammation and in the presence of high levels of TGF-β, there is mineralization in POF but not in PG. However, more studies are necessary to elucidate the role of cytokines and bone proteins in the metabolism of these lesions.
